# Prevalence, risk factors and mortality associated with colonic atresia: a population-based case-control study

**DOI:** 10.1007/s00383-025-06284-4

**Published:** 2025-12-23

**Authors:** Roni Kankaristo, Ilkka Helenius, Susanna Heiskanen, Johanna Syvänen, Teemu Kemppainen, Eliisa Löyttyniemi, Mika Gissler, Arimatias Raitio

**Affiliations:** 1 Department of Paediatric Surgery, Orthopaedics and Traumatology, University of Turku, Turku University Hospital, Turku, Finland; 2 Department of Orthopaedics and Traumatology, University of Helsinki, Helsinki University Hospital, Helsinki, Finland; 3 Department of Biostatistics, University of Turku and Turku University Hospital, Turku, Finland; 4 Department of Data and Analytics, Finnish Institute of Health and Welfare, Helsinki, Finland; 5 Department of Molecular Medicine and Surgery, Karolinska Institute, Stockholm, Sweden and Academic Primary Health Care Centre, Region Stockholm, Stockholm, Sweden

**Keywords:** Colonic atresia, Maternal risk factor, Congenital anomaly, Prevalence

## Abstract

**Purpose:**

This study aims to explore maternal and pregnancy-related risk factors for colonic atresia (CA) and assess the national total prevalence, mortality, and frequency of co-occurring anomalies of this rare malformation in 2004–2017.

**Methods:**

This case-control study involved 36 cases with congenital CA identified from several Finnish registers. All cases were identified based on the ICD-9/ICD-10 codes and classified based on co-occurring anomalies. Five controls without gastrointestinal congenital malformations matched for residence and time of conception (± 1 year) were randomly selected for each case. Maternal risk factors were analyzed with data from the same registers.

**Results:**

Total prevalence of CA was 0.45/10,000, birth prevalence was 0.37/10,000 and live birth prevalence was 0.36/10,000. The overall prevalence trend did not change (*p* = 0.11) during the study period. There were 15 (41.7%) isolated cases, 3 (8.3%) were associated with known syndromes and 18 (50.0%) had multiple congenital anomalies. Together there were 19.4% (*n* = 7) terminations or neonatal mortalities. An association was observed with maternal diabetes and CA (*p* = 0.03).

**Conclusion:**

The prevalence of CA in Finland is low with no significant change over the study period. Despite the high frequency of associated anomalies, the overall survival of CA is very high, 97%.

## Introduction

Colonic atresia (CA) is defined as a birth defect in which part of the colon is completely obstructed or missing [1]. CA can be further classified into four different subtypes using the classification by Grosfeld et al.: type I, involving mucosal atresia; type II, where the atretic ends are separated by a fibrous cord; type IIIa, in which a V-shaped mesenteric defect separates the atretic segments; type IIIb, also known as “apple-peel” atresia; and type IV, characterized by multiple atretic segments [2]. It is a very rare cause of intestinal obstruction and constitutes 2–15% of all intestinal atresias, with an estimated prevalence between 0.15 and 1 in 10,000 [3]. The pathogenesis of this anomaly is still unclear, but the most accepted theory is that a vascular insult due to volvulus, intussusception, hernia etc. causes damage to the fetal intestine [4, 5]. Genetics may also have a role in some cases [6, 7].

First symptoms of CA include swelling of the abdomen, inability to pass meconium and bilious vomiting [1]. Primary anastomosis and staged procedures with delayed anastomosis are the most used surgical procedures for treating CA [8, 9]. Most notable surgical complications are late adhesive bowel obstruction or leakage [9, 10].

CA has been reported to be slightly more predominant in males (M/F 4/3) [11], but more recent studies have also reported the prevalence to be equal between males and females [10]. CA can be either isolated or associated with other anomalies, such as other gastrointestinal malformations [12–16] like Hirschsprung disease [17–19] and extraintestinal malformations [20–23]. Associated anomalies have been reported in 33% [11, 24] of the cases. Potential risk factors for CA include maternal occupational exposure to ionizing radiation [25], gestational fever [26] and genitourinary infections [27]. However, due to the rare occurrence of CA, these risk factors have been challenging to study and only individual studies concerning the risk factors are available.

Etensel et al. reported an overall mortality due to CA as 25.7%. The most important factor affecting mortality was time from birth to operation, as cases with delay of more than 72 h have a considerably higher mortality than those operated within 72 h after birth [11]. Some studies have reported an overall survival of 100% [9, 10].

Against this background, the aim of this study was to explore maternal and pregnancy-related risk factors for CA and to assess the national total prevalence, mortality, and frequency of co-occurring anomalies of this rare malformation.

## Methods

All cases (*n* = 36) with major congenital CA born in Finland between Jan 1, 2004 and Dec 31, 2017 were identified from the Finnish Register of Congenital Malformations, the Medical Birth Register, and the Register on Induced Abortions, all maintained by the Finnish Institute for Health and Welfare. Data concerning the cases was collected for the first two years of life. Information on maternal prescription medicine use was obtained from the Register of Reimbursed Drug Purchases (Social Insurance Institution of Finland). These registers receive information based on a legally compulsory announcement request and have been validated as confirming accurate data with high coverage [28–30].

Annual birth rates were derived from The Medical Birth Register. Birth prevalence and total prevalence are given per 10,000 births, and live birth prevalence is given per 10,000 live births as defined by EUROCAT [31]. All cases with ICD-9 (751200 and 751280) and ICD-10 codes (Q42.8 and Q42.9) from 2004 to 2017 were identified and reviewed. Five controls without gastrointestinal congenital malformations from the Medical Birth Register matched for residence, maternal age, and time of conception (± 1 year) and their mothers were randomly selected for each case. Classification of associated syndromes, chromosomal abnormalities and anomalies and exclusion of minor anomalies was performed using EUROCAT guidelines [31].

This study used case-control design for risk factor analyses. Maternal risk factors in the register were analyzed including body mass index (BMI), parity, history of miscarriages, smoking, maternal chronic diseases, and prescription drug purchases. Diabetes group contained both type-1 and type-2 diabetes (ICD-10 codes E10, E11, O24.0, and O24.1) diagnosed before conception or during pregnancy and gestational diabetes defined as a recorded diagnosis of gestational diabetes (O24.4) and/or an abnormal glucose tolerance result.

Prevalence of CA in cases exposed to medicine and other risk factors was compared to prevalence of CA in unexposed cases by using χ^2^-test and Fisher´s exact test. Odds ratios (OR) along with adjusted odds ratios (aOR) with 95% confidence intervals (CI) were calculated for the risk caused by gestational medicine use. Change in livebirth prevalence over years was evaluated with linear regression. The analyses were performed using SAS System, version 9.4 for Windows (SAS Institute).

### Ethical considerations

The approval of the Finnish Social and Health Data Permit Authority Findata (permit number THL/6258/14.02.00/2021) and Turku University Hospital were obtained before conducting this study.

## Results

**Fig. 1:**
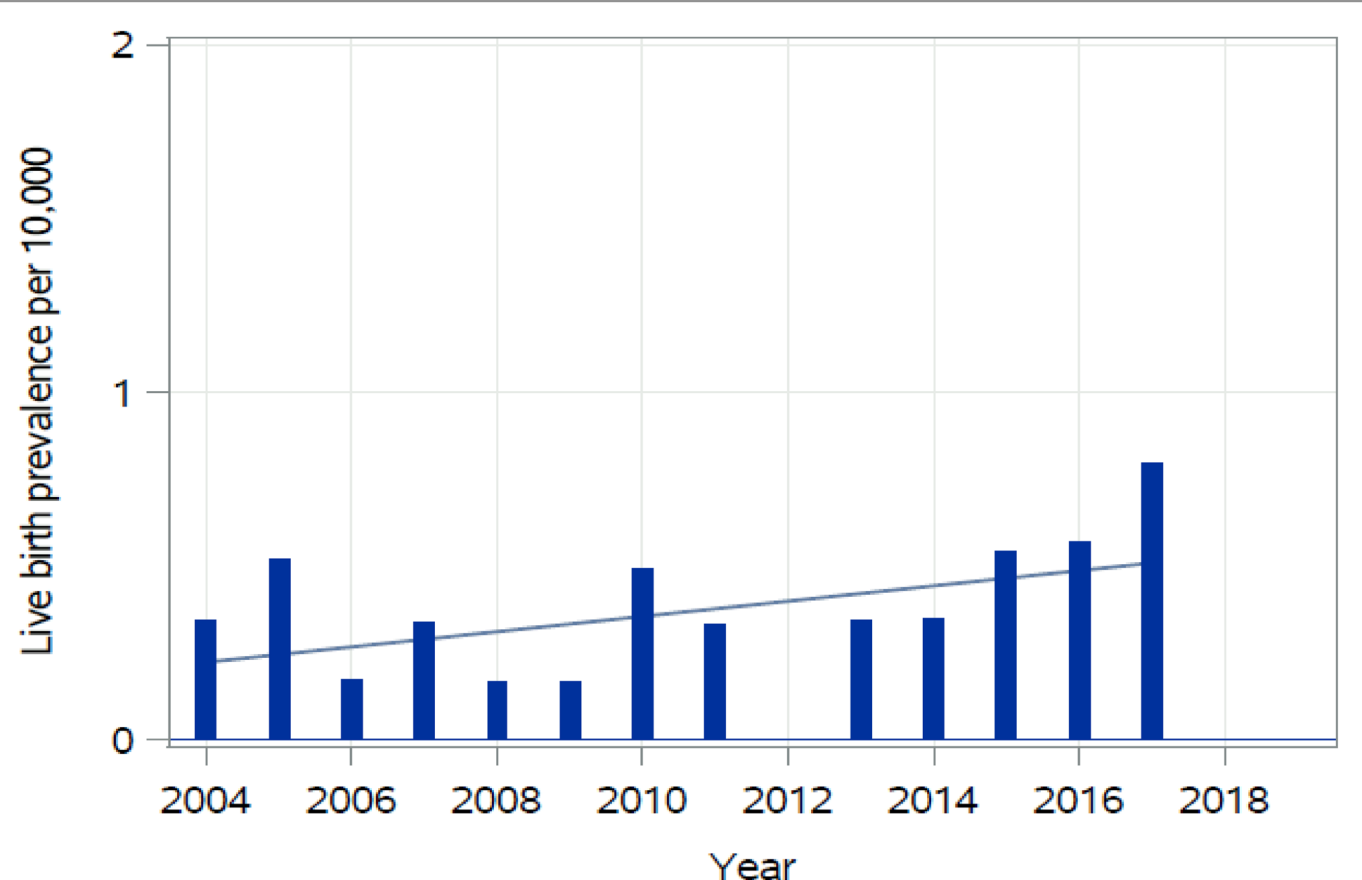
Live birth prevalence of colonic atresia. A linear fit is shown to illustrate the trend in the data

This study identified 36 CA cases in Finland between 2004 and 2017 including 29 (80.6%) live births and together 7 (19.4%) terminations of pregnancy for fetal anomaly or stillbirths. Of the 36 cases, 19 were females, 11 were males and 6 were unknown. Total prevalence of CA was 0.45/10,000, birth prevalence was 0.37/10,000 and live birth prevalence was 0.36/10,000. The overall prevalence trend was increasing but statistically insignificantly, *p* = 0.11 (Fig. 1). At 1 year 28 (97%) were alive.

There were 15 (41.7%) isolated cases, 18 (50.0%) had MCA (multiple congenital anomalies) and 3 (8.3%) were associated with known syndromes (Table 1). Other gastrointestinal defects were present in 15 (41.7%) cases, followed by urinary tract anomalies in 7 (19.4%) and limb deformities/defects in 6 (16.7%) of the cases. Chromosomal abnormalities were present in 3 (8.3%) of the cases (Table 2).

Mean maternal age was 29.3 years (SD: 6.47) Mean gestational age at birth was 38.6 weeks (SD: 2.58) and mean birth weight was 3148 g (SD 785, 5–95 percentile range 1450–4260 g). TOPFA (termination of pregnancy due to fetal anomaly) rate among all cases was 16.7% (*n* = 6), but in presence of syndromes it rose to 100%. In the presence of MCA, the TOPFA rate was 16.7%, and in isolated cases there were no terminations.

In risk factor analysis, an association was observed with maternal diabetes and CA (*p* = 0.03, OR 2.65, 95% CI 1.10–6.42). In the case group 10/30 (33.3%) mothers had diabetes, while in the control group, only 27/180 (15.0%) were diabetic. None of the other maternal risk factors are reported here as they were statistically insignificant (Table 3).

**Table 1:** Distribution of colonic atresias in presence of other comorbidities among birth status and terminations

	Isolated % (*n*)	Multiple CA % (*n*)	SDR % (*n*)
Live birth, *n* = 29 (80.6%)	48.3 (14)	51.7 (15)	− (0)
Stillbirths + terminations, *n* = 7 (19.4%)	14 (1)	43 (3)	43 (3)
All, *n* = 36	41.7 (15)	50 (18)	8.3 (3)

*SDR* association with a known syndrome, *CA* congenital anomaly.

**Table 2:** Incidence of individual concurrent anomalies among 36 colonic atresia cases organized by affected organ systems

Isolated % (*n*)	Nervous system % (*n*)	Heart % (*n*)	Cleft % (*n*)	Eye % (*n*)	Ear, face and neck % (*n*)	GI % (*n*)	Respiratory % (*n*)	Urinary % (*n*)	Genital % (*n*)	AWD % (*n*)	Limb % (*n*)	Spine % (*n*)	Chromosomal % (*n*)	Other anomalies/syndromes % (*n*)
33.3 (12)	< 8.3 (< 3)	8.3 (3)	< 8.3 (< 3)	< 8.3 (< 3)	< 8.3 (< 3)	41.7 (15)	< 8.3 (< 3)	19.4 (7)	< 8.3 (< 3)	8.3 (3)	16.7 (6)	< 8.3 (< 3)	8.3 (3)	< 8.3 (< 3)

Due to Finnish legislation, exact number of patients is not reported in frequencies < 3. *GI* gastrointestinal, *AWD* abdominal wall defects

**Table 3:** Univariate analysis for maternal risk factors

	Cases (total number of cases)	Controls (total number of controls)	Odds ratio	95% CI
BMI < 18.5	< 3 (< 30)	8 (167)	0.71	0.08–6.20
BMI 18.5-24.99	18 (< 30)	112 (167)	reference	reference
BMI ≥ 25	8 (< 30)	47 (167)	1.04	0.41–2.66
Smoking	5 (30)	26 (172)	1.27	0.37–4.32
Primiparity	14 (30)	93 (180)	reference	reference
Multiparity	16 (30)	87 (180)	1.25	0.53–2.97
No miscarriages	21 (30)	144 (180)	reference	reference
One miscarriage	5 (30)	27 (180)	1.87	0.57–6.15
Two or more miscarriages	4 (30)	9 (180)	3.17	0.84-12.0

The total number of cases varies due to missing data

## Discussion

Based on this population-based study CA is associated with high survival rates approaching 100%. More than half of the cases are associated with other congenital malformations and/or syndromes.

This register-based study comprising 36 CA cases from the years 2004 to 2017 observed a total national prevalence of 0.45/10 000 which is in line with the other reported studies [3]. There was a slightly increasing trend in prevalence, but this was not statistically significant.

Tahkola et al., in a study with partially overlapping periods, reported a median maternal age of 29 years and a median gestational age of 39 weeks for CA in a single center study from Finland, aligning with the findings of the current study. They also reported a median birth weight of 3200 g which is in line with the current study [10].

We observed an association with maternal diabetes and CA. Maternal diabetes has been known to increase the risk for other congenital malformations [32–35] and hyperglycemia is strongly associated as a primary teratogen that causes embryonic maldevelopment [36]. Because the maternal risk factors for CA are still uncertain, this finding is notable as only a handful of studies have observed risk factors for CA. Howley et al. noticed an association between maternal urinary tract infections and colonic atresia/stenosis from the data collected in the National Birth Defects Prevention Study (NBDPS) from 1997 to 2011 [27]. Waller et al. found an association between maternal cold or flu with fever and colonic atresia/stenosis from the same data [26]. Lim et al. observed an association between maternal occupational exposure to ionizing radiation and colonic atresia, with the exposure occurring between 3 months prior to conception and the end of the first trimester of pregnancy [25]. Guttman et al. in their study found that five patients with multiple intestinal atresias, including colonic, had common ancestors and proposed that in the case of multiple atresias a rare autosomal recessive gene might be the responsible agent [6]. Puri et al. also noticed identical histologic findings in their cases of hereditary multiple intestinal atresias [7].

More extensive research has been done on the maternal risk factors for other congenital anomalies. Maternal obesity, smoking, diabetes and exposure to organic solvents during pregnancy has been associated with an increased risk for congenital heart diseases [37]. Medications such as ACE-inhibitors, vitamin A/retinoic acid and folic acid antagonists are additional possible risk factors [38]. Low birth weight, prematurity and maternal smoking are associated with an increased risk for isolated limb reduction defects [39]. Additional research into the risk factors of CA and other congenital anomalies is very important for the future, as knowledge of these factors can improve pre and postnatal diagnostics of these diseases. Also, in some cases avoidance of certain medications or other risk factors can potentially reduce the incidence of some congenital anomalies.

In our study the most common associated anomaly was other GI anomalies which were present in 42% of cases. In other studies, the association of other GI anomalies has also been frequently reported with a rate varying between 20% and 80% of cases [10, 11]. Overall, there were 41.7% isolated cases in our study while others have reported some 60% of the CA cases to be isolated malformations [10, 11]. This finding may reflect the accuracy and coverage of the used register as all the information regarding anomalies from the first year of life is collected. Further, these registers are updated with new diagnoses even later and therefore contain associated anomalies diagnosed also after initial admission at birth. Screening and diagnosis of co-occurring anomalies is important, as Saha et al. concluded in their study that associated anomalies are an important factor that must be given attention to avoid mortality [40].

Etensel et al. reported a mortality rate of 25.7% in CA according to their literature review and almost 50% mortality in their own case series [11]. On the other hand, Tahkola et al. reported a 1-month survival of 100% between 1978 and 2019 in Finland [10], which is in line with 97% survival of infant period observed in the current study. However, all of the syndromic cases in the current study were terminated which may influence mortality positively. It should also be noted that the study by Tahkola et al. [10] might partly include some of the same cases as our study.

### Strengths and limitations

This retrospective study is based on high quality national registries with validated accurate, high coverage population-based data [28–30]. The number of cases in our study was also among the highest in any studies published until now. However, the small number of cases remains the greatest limitation of this report. This study also only relies on retrospective register data. The different types of CA could not be classified based on the register data.

In conclusion, the prevalence of CA in Finland is very low with no significant change over the study period. Despite the high frequency of associated anomalies, the overall survival of CA is very high, 97%.

## Data Availability

No datasets were generated or analysed during the current study.
